# Mixed Cryoglobulinemia Syndrome (MCS) due to untreated hepatitis B with uncommon presentation: case report and literature review

**DOI:** 10.1186/s41927-020-00159-y

**Published:** 2020-11-18

**Authors:** Nasam Alfraji, Vandan D. Upadhyaya, Christopher Bekampis, Halyna Kuzyshyn

**Affiliations:** 1 Department of Medicine, Jersey Shore University Medical Center, Neptune, NJ 07753 USA; 2 Internal Medicine Residency Program, Department of Medicine, Jersey Shore University Medical Center, Hackensack Meridian Health, Neptune, NJ 07753 USA; 3 Department of Rheumatology, Jersey Shore University Medical Center, Neptune, NJ 07753 USA

**Keywords:** Pulmonary alveolar hemorrhage, Mixed cryoglobulinemia, Vasculitis, Hepatitis B virus

## Abstract

**Background:**

The mixed cryoglobulinemia (MC) syndrome is a systemic inflammatory syndrome that causes small-to-medium vessel vasculitis due to cryoglobulin-containing immune complexes most commonly caused by chronic hepatitis C virus (HCV), and rarely by chronic hepatitis B virus (HBV). Its clinical presentation is significantly varied, with manifestations ranging from purpura, arthralgia, and myalgia to more severe neurologic and renal involvement. Pulmonary involvement as organizing pneumonia, alveolar hemorrhage, and pulmonary vasculitis have been reported, but appear to be quite rare.

**Case presentation:**

We report an uncommon case of a patient who presented with primary pulmonary syndrome without renal involvement in the setting of MC, due to untreated chronic hepatitis B infection. Early diagnosis and consequent institution of glucocorticoids, B-cell-depleting monoclonal antibody and antiviral therapy led to a favorable outcome and prevented any fatal sequelae.

**Conclusion:**

Pulmonary compromise in MC syndrome is very uncommon and carries a high rate of mortality. Therefore, in patients with HBV presenting with hemoptysis, physicians must carry a high clinical suspicion for alveolar hemorrhage secondary to cryoglobulinemic vasculitis.

## Background

Serum cryoglobulins are found in a wide variety of disorders [1]. However, majority of people with cryoglobulins can be asymptomatic and their presence can carry no clinical significance [1].

Symptoms and clinical findings are correlated with the underlying Brouet type of cryoglobulin - Type I, II, or III [1, 2]. Cryoglobulins type I are usually associated with lymphoproliferative disorders, while type II and III (mixed cryglobulins) are associated with infections, and connective tissue/autoimmune diseases [1–5].

Pulmonary involvement in MC is a rare but reported finding [6]. Alveolar hemorrhage has been noted in up to 3.2% of cryoglobulinemia cases, and most often been associated with hepatitis C antibodies [6–9]. Initially such cases were often mistaken for severe pneumonia, but persistent interstitial infiltrates and hemosiderin-laden macrophages in bronchoalveolar lavage fluid started to suggest otherwise [9]. Such features of pulmonary vasculitis are rarely seen in MC especially in correlation with untreated chronic hepatitis B infection [6–10]. Retrospective studies performed by Monti et al. on 717 mixed cryoglobulin patients only found 5.8% to have prevalence of HBsAg positivity [5]. While HBV affects more than 350 million people worldwide, cryoglobulinemic vasculitis can develop in only 1.2–4% patients infected with hepatitis B virus [10]. While reported to have glomerulus, skin, and liver involvement, HBV induced cryoglobulinemia presenting primarily with pulmonary alveolar hemorrhage is rarely documented in literature.

We report a rare case of mixed cryoglobulinemia syndrome due to untreated HBV infection presenting primarily with pulmonary finding without renal involvement.

## Case presentation

A 67-year-old Chinese male, chronic smoker, with past medical history of hypertension, asthma, and untreated hepatitis B presented to our emergency department (ED) with complaint of sudden onset of frank hemoptysis of 1 day duration. He reported that he had a productive cough with significant amount of blood and clots measuring about a cupful. He mentioned that his cough had been present for over a month but only now was present with frank blood. The patient also complained of generalized fatigue and endorsed losing over 15 pounds over the course of the last several months unintentionally. He denied any shortness of breath, chest pain, fever, chills, night sweats, epistaxis, dry eyes, dry mouth, vision changes, photosensitivity, oral ulcer, dysphagia, abdominal pain, nausea, vomiting, constipation, or diarrhea. He denied any urinary disturbance, muscle pain, joint pain or swelling, blood in urine or stool, and any Raynaud’s type symptoms. The patient had immigrated to United States about 20 years prior, with a questionable history of treated tuberculosis about 20 years ago, and no recent travel history. He endorsed a family history only significant for lung cancer in his father. He reported drinking 1–2 alcoholic beverages every day and smoking one pack of cigarettes for the past 20 years.

Few days prior to this admission, patient had presented to our ED with complaints of bilateral lower extremity and upper extremities numbness, and rash that has started 1 month prior. The rash at the time was described as a palpable purpura over the lower extremities. The patient denied any associated joint pain or joint swelling at that time. He was discharged from the ED with a short course of prednisone, and the rash improved.

In the ED, the patient’s vitals were blood pressure 127/72 mmHg, pulse 60/min, temperature 98.3 F, respiratory rate 14 breaths/minute, and pulse oximetry 98% on room air, and he was not in need of any supplemental oxygen. On physical exam, patient was not in acute distress. He was able to answer questions appropriately, alert and oriented to time, place and person. Head and neck examinations were unremarkable for lymphadenopathy or Jugular venous distension (JVD). Oral examination showed blood spots that were noted on the roof of the mouth and the base of the tongue and poor oral dentition, but no ulcerations or any other lesions found. On chest auscultation, scattered coarse crackles were noted and decreased breath sound in the right and left middle and lower fields. Cardiology, gastrointestinal, and neurological examinations were unremarkable. No joint tenderness or swelling appreciated at the musculoskeletal examination. Skin examination revealed dark red non-blanching rash on the bilateral shins and calves.

Initial complete blood count and complete metabolic panel were all within normal limits (Table 1, column 2). Erythrocyte sedimentation rate (ESR) initially was 13 mm/hr., and on subsequent follow up increased to 44 mm/hr. C-reactive protein (CRP) was elevated at 21.9 mg/L. Urinalysis was negative, with no protein or cells. Hepatitis C antibody negative. Hepatitis B surface antigen was positive, and viral load detected by quantitative nucleic acid amplification test (NAAT) at 530 IU/mL. Coagulation profile was within normal (INR: 0.9, activated PTT: 31). Infectious work-up over the next subsequent days was found negative including for tuberculosis.

**Table 1:** Summary of laboratory investigations at baseline and follow-ups

Lab value	Baseline	1st admission	2nd admission	3 months after starting treatment	Reference value
BUN	12	8	10	9	5–25 mg/dl
Creatinine	0.72	0.87	0.87	0.90	0.61–1.24 mg/dl
GFR	> 60	> 60	> 60	87	> 60
AST	19	28	32	18	10–42 U/L
ALT	12	18	27	12	10–60 U/L
ALP	77	82	69	103	38–126 U/L
INR	1.00	0.92	0.96	–	0.88–1.15
PTT	32	32	31	–	26–39 s
WBC	10.3	6.6	6	6.3	4.5–11.0 K/uL
Hemoglobin	13.0	14.6	13.4	15.4	13.2–17.5 g/dl
Platelets	163	203	187	213	140–450 K/uL
ESR	19	13	44	20	0–15 mm/h
CRP	–	21.9	–	2	0–10 mg/L
C3	–	54.8	–	87	85–170 mg/dl
C4	–	< 2	–	7	16–40 mg/dl
RF	–	2460.0	–	–	< 20 U/mL
c-ANCA	–	< 1:20	–	–	< 1:20
p-ANCA	–	0	–	–	0–19 U/mL
Cryoglobulin screen	–	Positive	–	Positive	Non-detected
Cryoprecipitan IFE	–	Type II	–	Type II	–
IGA cryprecipitate	–	15	–	–	0 mg/dl
IGG cryprecipitate	–	97	–	–	0 mg/dl
IGM cryprecipitate	–	109	–	–	0 mg/dl
QuantiFERON TB	–	Negative	–	–	–
Hep B surface Ag	–	Positive	–	Positive	–
Hep B core IgM Ab	–	Negative	–	Negative	–
Hep B surface Ab	–	Negative	–	Negative	–
Hep B NAAT		530 IU/mL	–	Undetected	–
Hep C Ab	–	Negative	–	–	–
Hep A Ab IgM	–	Negative	–	–	–
HIV 1 & 2 Ag/AB	–	Negative	–	–	–

Further work-up (Table 1, column 2) was performed during the admission and yielded highly elevated rheumatoid factor (2460.00 U/mL), reduced complement levels (C3 54.8, C4 < 2).

**Fig. 1:**
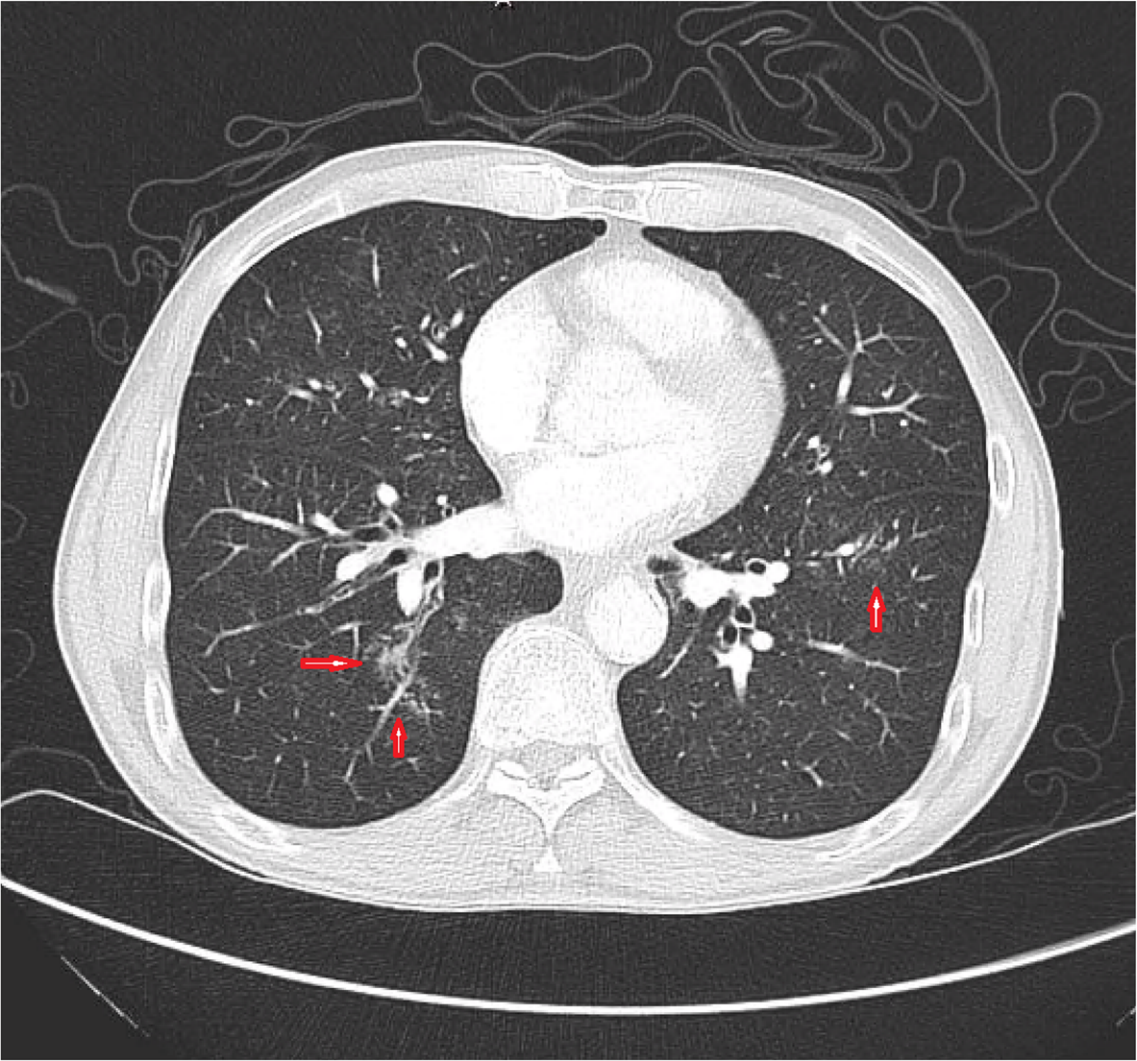
Computed tomography of chest revealing centrally located ill-defined ground-glass and mixed density nodular opacities greater on the right side

A computed tomography angiography of the chest showed no evidence suggestive of pulmonary embolus but was suggestive of ground-glass and mixed density nodular opacities with increased bilateral peri-bronchial thickening with tracheal and bronchial mucous stranding and increased right middle lobe mucous plugging indicating chronic low-grade inflammatory process (Fig. 1).

At that time, most of serology work-up were pending including cryoglobulins level and anti-neutrophil cytoplasmic antibody (ANCA), therefore patient was started on oral Azithromycin 500 mg daily, and intravenous Ceftriaxone 1 g daily for 7 days for pneumonia concern. His hemoptysis resolved during his hospital stay and hemoglobin remained stable. He was also initiated on antiviral therapy for hepatitis B. The patient was discharged home on empiric azithromycin for outpatient treatment for presumable pneumonia and follow-up bronchoscopy while waiting for serology.

One day after discharge, patient experienced another episode of hemoptysis, was readmitted for closer monitoring and further evaluation. At that time, the remaining serology (Table 1, column 2) was resulted revealing elevated cryoglobulins IgG and IgM level, and immunofixation electrophoresis of the cryoprecipitate disclosed a Type II Cryoglobulinemia. Patient was found negative for serine PR3 (protease3)-ANCA and MPO (myeloperoxidase)-ANCA.

**Fig. 2:**
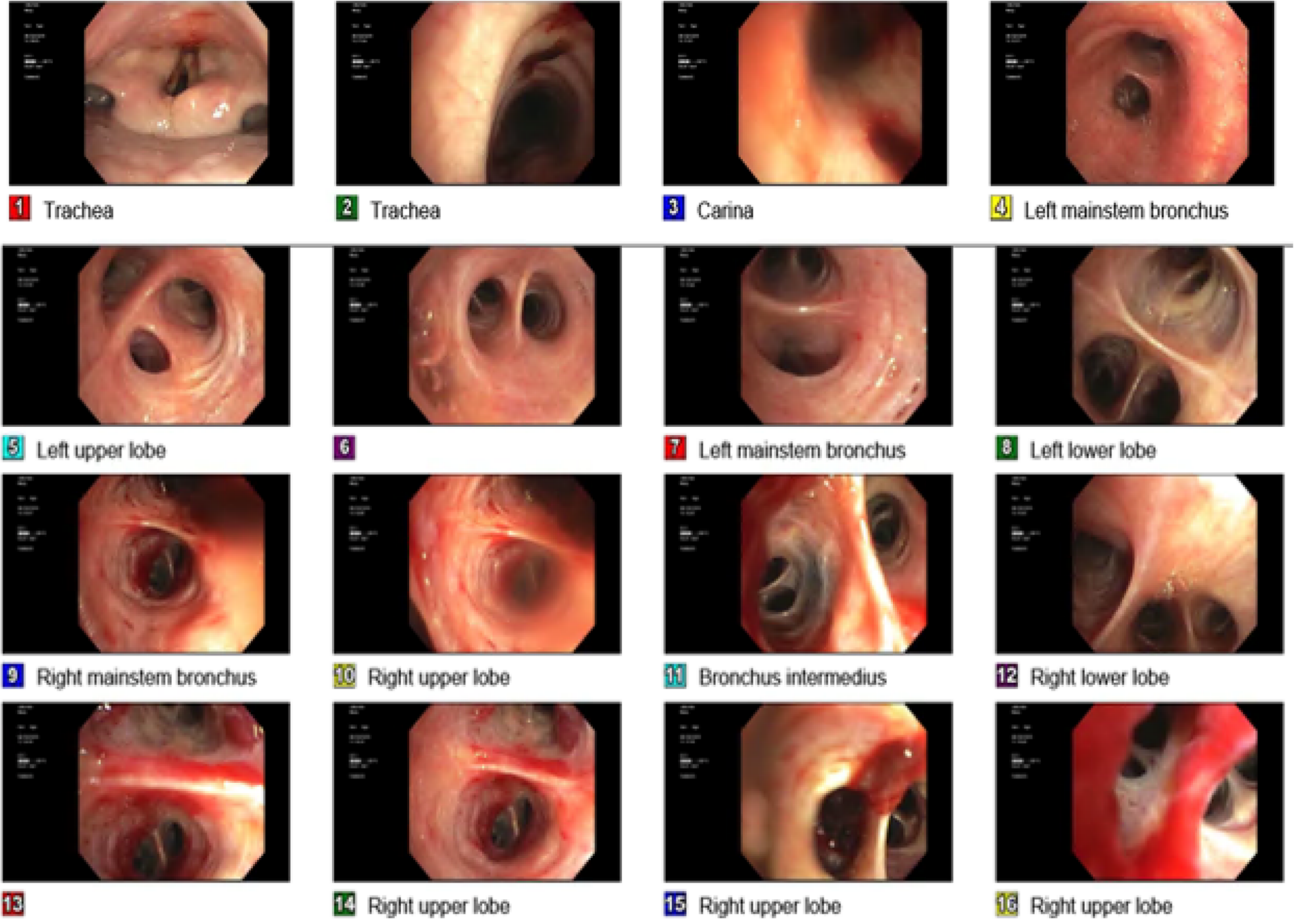
Pictures during bronchoscopy showing a friable and necrotic bronchial mucosa in the right upper segments with blood clots located in the right upper lobe

The patient underwent bronchoscopy (Fig. 2) at the 2nd hospital admission, which revealed a friable and necrotic bronchial mucosa with blood clots in the right lung, but no active bleeding was noticed. Analysis of the bronchoalveolar lavage (BAL) revealed no malignant cells, and no fungal elements identified, but few bronchial epithelial cells and hemosiderin-laden macrophages on a hemorrhagic background confirming the suspected diagnosis of alveolar hemorrhage. Bronchoalveolar lavage cultures were negative.

Therefore, the patient was diagnosed with cryoglobulinemic vasculitis and started on high dose oral corticosteroid, 60 mg Prednisone daily. His hemoptysis resolved, and kidney function remained stable. He was stable for discharge to follow up outpatient with rheumatologist for closer monitoring and to start Rituximab as an outpatient. The patient was continued on Entecavir for hepatitis B treatment under the follow up of hepatologist. He received Rituximab intravenous infusion of 1000 mg on week 0 and 2 (for total of two doses). Prednisone was tapered off on weekly basis over the following 3 months. With the treatment, hemoptysis resolved, purpuric skin rashes cleared, kidney function remained stable with no active urinary sediment, and C3, CRP, and ESR normalized. Hepatitis B viral load became undetectable on treatment with Entecavir.

## Discussion and conclusion

Cryoglobulinemia is a rare disease characterized by presence of cryoglobulins, which are serum immunoglobulins that precipitate at low body temperature and re-dissolve with rewarming [11]. There are three types of cryoglobulins according to Brouet et al., types I, II, and III [2]. Type I is associated with lymphoproliferative diseases, while type II, and type III, called mixed cryoglobulinemia (MC), are associated with chronic infections mainly chronic hepatitis C virus (HCV) and less commonly chronic HBV infections, and sometimes with autoimmune diseases [11].

Type I cryoglobulins can precipitate at cold exposure resulting in hyper-viscosity and sludging, and patients present with Raynaud’s phenomenon, digital ischemia, livedo reticularis, and central nervous system involvement [12]. Type II, and type III cryoglobulins (mixed cryoglobulins) can form immune complexes causing small to medium vessel vasculitis (Cryoglobulinemic vasculitis) in multiple tissues and organs [11, 12]. HCV-associated Cryoglobulinemic vasculitis (CV) is noted in 90–95% of cases, while hepatitis B virus-related Cryoglobulinemic vasculitis occurs in approximately 3% of cases only [11]. Patients with mixed cryoglobulinemia can present with ‘Meltzer’s triad” of purpura, arthralgia, and weakness to more serious manifestations with skin, neurological, renal, and rarely pulmonary involvement [12].

Pulmonary manifestations have been reported, but appears to be very rare, and ranging from shortness of breath, interstitial lung fibrosis, hemoptysis, and/or diffuse alveolar hemorrhage to respiratory failure [13, 14]. While glomerulonephritis is noted in up to 90% of cases with MC, alveolar hemorrhage occurs in approximately 3.2% of patients with mixed cryoglobulinemia [14].

Ferri et al. studied the clinical manifestations of 231 patients with mixed cryoglobulinemia, but 21 patients (9.1%) were lost to follow- up at the end of the study. Mild exertional dyspnea was noted in 15 and 26% of patients at the beginning and end of follow-up, respectively [15]. However, only 2% (four of 210) of patients had clinical/radiological evidence of interstitial lung involvement and only one patient was found to have hemoptysis. Of note, 92% of cases had hepatitis C virus (HCV) infection, whereas hepatitis B virus (HBV) considered the cause in only 1.8% of patients in that study [15]. Bombardieri et al. [16] tested 23 MC patients with lung function studies. Of these, 20 had minimal to absent respiratory symptoms and, of those with severe respiratory symptoms, only one presented with hemoptysis. Noticeably, 18 of 23 patients had radiographic evidence of interstitial lung disease. Amital et al. [7] studied 125 patients hospitalized with cryglobulinemia over a 23-year period at their center. Of these, only four patients (3.2%) developed alveolar hemorrhage.

Trejo et al. had 7043 patients tested for circulating cryoglobulins, 443 (6.29%) patients had a cryocrit of 1% or more [4]. Of the 443, 206 (47%) had clinical manifestations attributable to cryoglobulinemia during the progression of the disease. Pulmonary involvement was noted in 6 (1%) patients at onset of the disease, and in 9 (2%) During evolution of the disease [4]. A cohort study performed by Ramos-Casals et al. analyzed 209 patients with cryoglobulinemic vasculitis. Twenty-nine (14%) patients had life-threatening cryoglobulinemic vasculitis [17]. Of the 29, four patients had pulmonary hemorrhage. All had dyspnea, fever, hemoptysis, and pulmonary infiltrates on chest radiograph. The four patients died with no remarkable differences in the therapeutic regimens received by patients who died during the first episode and those who survived. Retamozo et al. study analyzed 279 HCV patients with cryoglobulinemia [18]. Pulmonary hemorrhage noted in 18 (6.4%) patients. Among these 18 patients, pulmonary presentations ranging from respiratory failure in 11 (61.1%) patients, hemoptysis in 9 (50%), and dyspnea in 6 (33.3%) patients. Thirteen (72%) patients had concomitant glomerulonephritis [18].

As there are few reported cases of HBV-related cryoglobulinemic vasculitis, there are still no definitive treatment guidelines issued yet [11]. According to Mazzaro et al. study published in 2016, a mono-therapy with antiviral agent nucleotide such as Lamivudine, Adefovir Dipivoxil, Entecavir, Telbivudine, or Tenofovir resulted in an excellent outcome in terms of viral clearance and clinical remission in HBV-related cryoglobulinemic vasculitis [19]. While corticosteroid therapy was able to treat the clinical symptoms of vasculitis, it failed in suppression of HBV viremia and/or treating immuno-logical features as stated in Mazzaro et al. study [19]. In Terrier et al. study, the use of corticosteroids and/or immunosuppressive agents with the lack of anti-viral agents were associated with refractory cryoglobulinemic vasculitis [20]. However, rituximab in combination with antiviral agents led to complete and sustained clinical remission in patients with refractory or relapsing HBV-related cryoglobulinemic vasculitis per Terrier et al. [11, 20].

Therefore, HBV-induced MC with moderate to severe manifestations (eg, glomerulonephritis, cutaneous ulcers, progressive neuropathy, diffuse vasculitis including pulmonary and central nervous system vasculitis), similarly to HCV-associated MC, can be treated with antiviral therapy, glucocorticoids, and rituximab [12, 20–22]. And the role of immunosuppressive therapy such as rituximab is to sustain remission, decrease the cumulative glucocorticoid dose usage, and prevent any dramatic deterioration in a disease that studies showed only 22% survival in patients who presented with pulmonary hemorrhage [11, 18, 20].

Our unique case presented with a rare phenomenon of hemoptysis and alveolar hemorrhage as an initial presentation of cryoglobulinemic vasculitis without renal involvement and in the setting of unusual association with untreated chronic hepatitis B infection. Therefore, physicians must always carry high index of suspicion for cryoglobulinemic vasculitis when patients with HBV present with hemoptysis/alveolar hemorrhage after excluding other causes such as infectious or neoplastic diseases. Bilateral lung infiltrates in such patients should warrant further investigation with bronchoscopy, as pulmonary hemorrhage on BAL is strongly suggestive of vasculitis [19]. As our case revealed, early bronchoscopy and diagnosis with a prompt treatment with corticosteroids, immunosuppressive agents (Rituximab), and antiviral therapy (Entecavir) resulting in a favorable outcome and improved patient survival. Our decision to start rituximab was on basis of the effectiveness of this option in patients with HCV-associated MC with moderate to severe signs of systemic vasculitis which our case can be considered under this category.

On our literature review, pulmonary vasculitis with primary hemoptysis caused by mixed cryoglobulinemia reported few times either in case or cohort studies as far as we noticed. Most of these cases had renal involvement with glomerulonephritis and were associated with HCV infection [13]. Most of these cases were treated with pulse methylprednisolone with other immunosuppressive therapy such as cyclophosphamide, rituximab, azathioprine, plasmapheresis, and commencing antiviral therapy [13]. However, pulmonary vasculitis and hemorrhage still have very poor prognosis despite appropriate management [23].

Due to the rarity and poor prognosis of pulmonary hemorrhage in mixed cryoglobulinemia, as well as the unusual association with hepatitis B infection, physicians must have a high index of suspicion for the disease to commence early treatment and reduce patient morbidity.

## Data Availability

Data sharing is not applicable to this article as no datasets were generated or analyzed during the current study.

## Abbreviations

HBV: Hepatitis B virus
HCV: Hepatitis C virus
ED: Emergency department
JVD: Jugular venous distension
ANCA: Antineutrophil cytoplasmic antibody
MPO: Myeloperoxidase
BAL: Bronchoalveolar lavage
MC: Mixed cryoglobulinemia
CV: Cryoglobulinemic vasculitis
NAAT: Nucleic acid amplification test
ESR: Erythrocyte sedimentation rate
CRP: C-reactive protein
PR3: Protease3
